# Long-Term Psychological Impact of the COVID-19 Pandemic on Healthcare Workers

**DOI:** 10.1192/j.eurpsy.2025.1270

**Published:** 2025-08-26

**Authors:** A. Gaddour, R. Nakhli, A. Chouchane, M. Bouhoula, K. Ben Amor, I. Jammeli, M. Bouhlel, I. Kacem, S. Chatti

**Affiliations:** 1 Occupational Medicine Department, Univerisity of Sousse, Ibn Jazzar University Hospital, Kairouan; 2 Department of Occupational Medicine, Faculty of Medicine of Sousse, Ibn Jazzar University Hospital, Tunis; 3 Occupational Medicine Department, University of Sousse, Farhat Hached University Hospital; 4 Department of Occupational Medicine, Faculty of Medicine of Sousse; 5 Department of Occupational Medicine, Faculty of Medicine of Sousse, Ibn Jazzar University Hospital, Sousse, Tunisia

## Abstract

**Introduction:**

The COVID-19 pandemic has placed immense psychological pressure on healthcare workers (HCWs) worldwide. In addition to the physical risks of infection, HCWs have faced high levels of anxiety due to their proximity to the virus, overwork, and the challenges of providing care in an ever-evolving environment. These psychological effects are expected to persist well beyond the initial phase of the pandemic.

**Objectives:**

This study explores the long-term impact of COVID-19 on both anxiety and depression among HCWs and identifies the main contributing factors.

**Methods:**

This cross-sectional study was conducted among HCWs infected with SARS Cov-2 between January and August 2022. Sociodemographic and professional data were extracted from participants’ medical records. Anxiety and depression were evaluated using the GAD-7 (Generalized Anxiety Disorder) and PHQ-9 (Patient Health Questionnaire) scales, respectively, through phone interviews conducted at least one year after their last COVID-19 infection

**Results:**

This study included a total of 184 healthcare workers (HCWs). The mean age of the participants was 41.93 years (± 8.6), with 95.1% being over 30 years old. Women comprised 81.4% of the cohort, and the majority of participants were nurses (45.7%), followed by administrative staff (20.1%). The median occupational seniority was 16 years. Notably, 28.3% of HCWs were employed in COVID-19 wards. Anxiety symptoms were reported by 92.9% of participants, with 17.3% experiencing mild anxiety, and 38.5% severe anxiety. Depression was prevalent in 69.1% of HCWs, with 27.2% presenting mild symptoms, and 24.5% experiencing moderate to severe depression. Female HCWs and those aged over 40 were significantly more likely to report symptoms of anxiety and depression (p < 0.001). Furthermore, HCWs with pre-existing conditions, such as diabetes, as well as those directly exposed to COVID-19 patients, exhibited significantly higher anxiety and depression scores. Nurses demonstrated particularly elevated levels of psychological distress, especially those working in COVID-19 wards.

**Conclusions:**

The COVID-19 pandemic has had a profound and lasting psychological impact on healthcare workers, with anxiety and depression levels remaining elevated long after the acute phase of the crisis. This study emphasizes the need for targeted psychological support interventions. Addressing the mental health needs of HCWs is crucial not only for their well-being but also for ensuring the ongoing efficacy and resilience of the healthcare workforce in times of crisis. Further research is warranted to explore long-term impacts and effective strategies for mental health support in this population.

**Disclosure of Interest:**

None Declared

